# Reporter Gene Silencing in Targeted Mouse Mutants Is Associated with Promoter CpG Island Methylation

**DOI:** 10.1371/journal.pone.0134155

**Published:** 2015-08-14

**Authors:** Julia V. Kirov, Michael Adkisson, A. J. Nava, Andreana Cipollone, Brandon Willis, Eric K. Engelhard, K. C. Kent Lloyd, Pieter de Jong, David B. West

**Affiliations:** 1 Children’s Hospital Oakland Research Institute (CHORI), Oakland, CA, United States of America; 2 Mouse Biology Program, University of California Davis, Davis, CA, United States of America; Thomas Jefferson University, UNITED STATES

## Abstract

Targeted mutations in mouse disrupt local chromatin structure and may lead to unanticipated local effects. We evaluated targeted gene promoter silencing in a group of six mutants carrying the tm1a Knockout Mouse Project allele containing both a LacZ reporter gene driven by the native promoter and a neo selection cassette. Messenger RNA levels of the reporter gene and targeted gene were assessed by qRT-PCR, and methylation of the promoter CpG islands and LacZ coding sequence were evaluated by sequencing of bisulfite-treated DNA. Mutants were stratified by LacZ staining into presumed Silenced and Expressed reporter genes. Silenced mutants had reduced relative quantities LacZ mRNA and greater CpG Island methylation compared with the Expressed mutant group. Within the silenced group, LacZ coding sequence methylation was significantly and positively correlated with CpG Island methylation, while promoter CpG methylation was only weakly correlated with LacZ gene mRNA. The results support the conclusion that there is promoter silencing in a subset of mutants carrying the tm1a allele. The features of targeted genes which promote local silencing when targeted remain unknown.

## Introduction

Random integration of foreign DNA into mammalian genomes is known to provoke a response resulting in histone modification, and marked by DNA methylation at CpG dinucleotide sites, with the end result being the silencing of any potential transcriptional elements. This silencing is particularly effective against repeat elements [1] and retrotransposon sequences [2]. However, since the degree of silencing depends upon the site of insertion, local chromatin organization and features also must play a role. Silencing has been problematic in the construction of vectors for random transgene insertion and expression in the creation of animal models since vectors are often inserted as concatemers and provoke silencing [3]. Furthermore, the potential for silencing of viral sequence is an important consideration for developing strategies for gene therapy [4]. Silencing of engineered transgenes has been mitigated by avoiding viral repeat elements known to provoke silencing [4], by engineering into the vector flanking insulators of DNA sequence to reduce local effects of the region on the transgene [5–6], and also by targeting the transgene to genomic regions thought to be less responsive to the presence of foreign DNA, e.g., the Rosa26 locus in mice [7–8].

The majority of gene targeting experiments in mammalian systems are designed to eliminate function of targeted genes, although many “knockins” have been designed to introduce specific mutations, or to express alternative sequences under the control of the native gene promoter. Often targeting vectors contain reporter sequence such as bacterial beta-galactosidase (LacZ) or green fluorescent protein, as well as selectable markers such as a neomycin resistance cassette in order to facilitate mutant selection in stem cell populations [9]. Since the majority of these gene targeting events are designed to knock out, or eliminate gene expression, then the consequences of silencing has been thought to be manageable, except that silencing of vector elements in the stem cells might interfere with selection of targeted cells using antibiotic resistance, or cause silencing of a reporter gene in the adult mutant. Strategies have been developed to eliminate most of the foreign DNA from targeting vectors after genomic integration by engineering recombinase sites flanking the selection cassette allowing the removal of vector components at any stage in the production of the model [10–11].

An earlier report in mouse studied the silencing of a randomly integrated transgene containing LacZ driven by a ubiquitous promoter. Silencing of transgene expression was correlated with the CpG content of the LacZ sequence in an allelic series of random integrants [12]. In that report, decreasing CpG content of the LacZ sequence was correlated with decreased methylation of CpGs in the heterologous promoter and reduced silencing assessed by enzyme activity. These data suggest that the CpG content of reporter genes, or other elements in transgenes, may have important local effects.

Recent large-scale mutagenesis programs in mice have created a valuable resource for studying the consequence of loss of function mutations in mammalian systems. Programs such as the European Mouse Disease Clinic (EUMODIC; www.europhenome.org; [13]); the Sanger Center Mouse Genetics Project [14]; KOMP knockout projects [15], and other programs recently organized as the International Knockout Mouse Consortium (www.mousephenotype.org), are producing thousands of targeted loss-of-function mutations in mouse protein-coding genes. Many of the targeting vectors contain both a reporter gene (LacZ) along with a neomycin resistance selectable marker. These mutations will provide a valuable resource for studying gene function, but they also provide a remarkable resource for studying the unintended consequences of targeting such as silencing of the targeted gene, or effects on the expression of neighboring genes. Since the targeting vector sequence is constant, while the location and local environment changes with each targeting event, then it may be possible to use this resource as a tool to better understand and characterize the mechanisms provoking transgene silencing and by inference gene regulation.

In a pilot study characterizing LacZ reporter gene expression in KOMP mutants [16] we noted that a subset of mutants had no LacZ staining despite gene expression surveys indicating the gene was expressed at moderate to high levels in some tissues. This suggested to us that these mutations may not be staining for LacZ due to promoter silencing of the targeted gene. In order to assess this, and preliminarily evaluate if DNA methylation marks the silenced genes, we identified a set of KOMP mutants with expected patterns of LacZ staining, and another set of mutants with no LacZ staining although it was expected based upon other gene expression surveys. In these two sets of mutants, we evaluated promoter CpG island methylation, LacZ coding sequence methylation, and quantified mRNA of the targeted gene and the LacZ reporter in order to determine if gene silencing was a possible explanation for the lack of reporter gene staining.

## Materials and Methods

### Mouse Production

Homozygous mutant C57BL/6N mice, and wild type controls, created from Knockout Mouse Project (KOMP) targeted stem cells [9], were studied. The mutant allele was either the KOMP CHORI-SANGER-DAVIS (CSD) “Knockout First” conditional-ready allele or a CSD “deletion” mutant where the recombination removed the critical exon. These alleles are gene traps and carry a LacZ reporter gene driven by the targeted gene promoter, along with a Neo selection marker driven by a heterologous promoter. For a description of these alleles, visit the website of the International Mouse Phenotyping Consortium (IMPC: www.mousephenotype.org). Targeting in the mice was confirmed by long-range PCR and zygosity by qPCR of LacZ coding sequence [17]. Homozygous Mutants, and Wild Type control (WT, Control) mice, were produced by heterozygous breeding of mutants and therefore shared a common C57BL/6N background strain and were reared in identical environmental conditions. Pups were weaned at ~21 days of age, and maintained on *ad libitum* Harlan Teklad Global Rodent Diet #2918 and water, in an environmentally controlled facility with a 12:12hr light:dark cycle. Mice were euthanized at ~7 weeks of age under isoflurane anesthesia with a thoracotomy and cervical dislocation. Tissues were rapidly harvested and quick frozen in liquid nitrogen, and stored at -80degC until used. All animal work followed the Guide for the Care and Use of Laboratory Animals of the Institute of Laboratory Animal Research of the National Institutes of Health and was approved by the Institutional Animal Care and Use Committee at the University of California, Davis.

We selected six mutant lines for study based upon LacZ staining pattern in adult mutants, gene expression patterns described in two gene expression atlases, and the presence of CpG islands (CGI) in the promoter of the targeted gene. These mutant lines were selected from a pool of ~90 mutant lines that were part of a pilot study of 313 mutant lines with LacZ staining, for which we had also banked frozen tissue samples. It was noted that three of the mutant lines had no LacZ staining even though it was expected based upon gene expression atlases using Affymetrix Chip technology. We identified three mutant lines from the same set of ~ 90 lines, with similar gene expression data in these other atlases which did have LacZ staining. The LacZ staining pattern for these mutants is reported at: www.kompphenotype.org [16]. Presence of CGIs was determined from analyses of mouse genomic DNA sequence using the UC Santa Cruz Genome Browser (http://genome.ucsc.edu/index.html; [18]) with the criteria defining a CGI of: a minimum length of 200bp, a minimum GC content of 50% and observed-to-expected CpG ratio greater than 60%. Tissue expression patterns were assessed by consulting two mouse expression atlases in the BioGPS database (www.biogps.org; [19]) and the Gene Expression Omnibus (GEO; http://www.ncbi.nlm.nih.gov/geo/) available at the National Center for Biological Information. We identified three mutant lines (Lyplal1^tm1a(KOMP)Wtsi^, Rab32^tm1a(KOMP)Wtsi^, Rgcc^tm1(KOMP)Wtsi^) with apparent reporter silencing (Silenced) based upon the lack of LacZ staining in mutant tissues despite expression data-bases reporting moderate to high expression of the native gene. Three mutant lines were identified (Ninj1^tm1a (KOMP)Wtsi^, Dstn^tm1a(KOMP)Wtsi^, Arap1^tm1a(EUCOMM)Wtsi^) with LacZ staining as predicted by the gene expression atlases (Expressed). Four of these alleles were the “Knockout First” allele, the *Rgcc* mutant was a deletion mutant where the critical exon was deleted, and the *Dstn* mutant carried a promoterless neo selection marker. All of these targeted genes had CpG islands in the promoter. See S1 Table for a list of mutants and tissues, and a summary of the gene expression data from BioGPS and GEO. For the Silenced Group of mutants we analyzed tissues with the highest gene expression according to the databases. For the Expressed group of mutants we analyzed 7 tissues where available including: brain, spleen, heart, kidney, liver, lung, muscle in order to evaluate the same tissues as evaluated in the Silenced group. Plots of CpG islands in the promoters of these genes are provided in S1 Fig. For each mutant line, and wild type controls, RNA and DNA was isolated and processed from 3 homozygous male mice.

### Experimental Design

**Fig 1:**
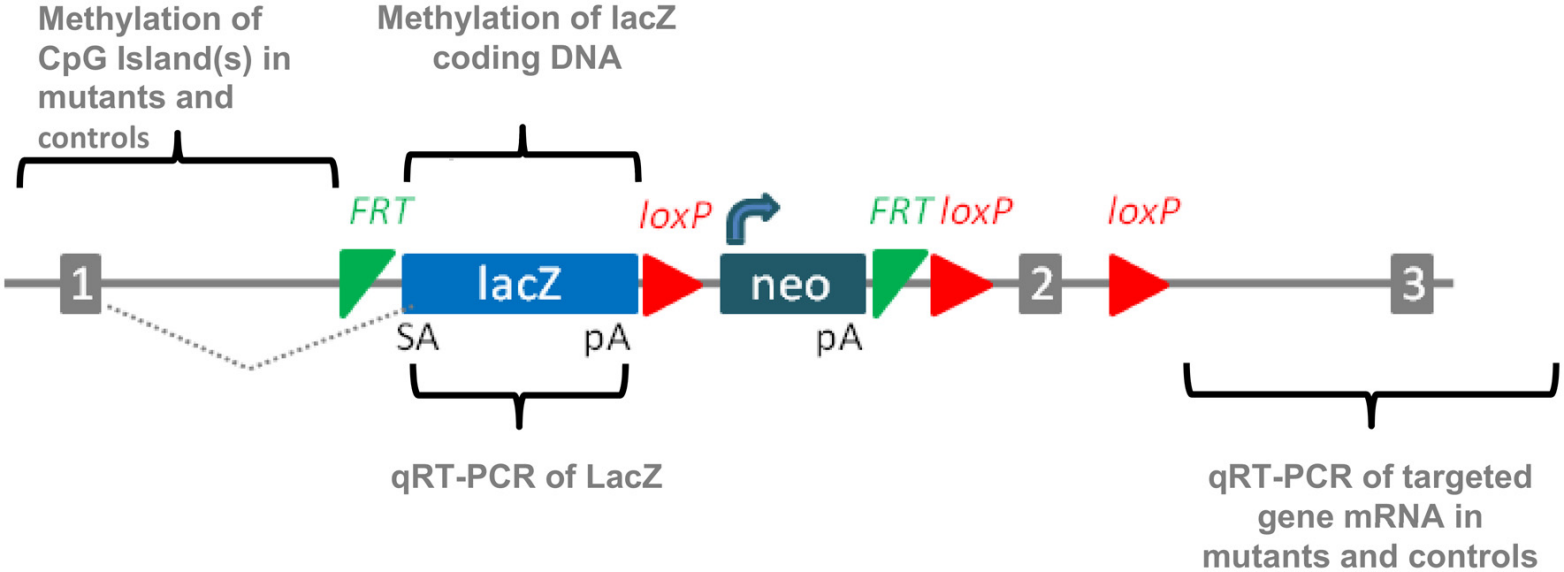
Schematic of Experimental Approach. A cartoon of the knockout first allele is shown with exons indicated by gray blocks numbered 1–3. The targeted exon in this schematic is #2, with the targeting vector replacing that critical exon with an identical exon flanked with LoxP sites. Proximal to the critical exon are placed in tandem a LacZ reporter gene driven by the targeted gene promoter, followed by transcriptional stop and polyadenylation signal, and then followed by a heterologous promoter driving a neomycin resistance gene. The gene regions evaluated for expression by qRT-PCR and/or for methylation by sequencing bisulfite treated DNA are indicated in brackets.

We used qRT-PCR to assess mRNA levels of each gene in wild type control and mutant mice, and to measure the mRNA of the LacZ reporter gene in mutants. By sequencing bisulfite-treated DNA, we assessed DNA methylation of CpG islands found in the promoter regions of each targeted gene, and methylation of the LacZ reporter gene coding sequence in mutants. Fig 1 presents a schematic of the gene structure and the DNA sequences assayed for CpG methylation and for quantitating expression by qRT-PCR.

### RNA Isolation and Quantitative RT-PCR

Tissue RNA isolation was performed using TRI-reagent (Sigma, St. Louis, MO) according to the manufacturer’s protocol. To ensure homogeneous disruption, tissue samples weighing 30-60mg were homogenized in 1ml of TRI-reagent using a bead mill at 30Hz for 45sec x2. Heart and muscle were ground to a fine powder with a mortar and pestle on dry ice prior to bead mill homogenization. Phase separation was achieved using 0.2mL of chloroform followed by isopropanol precipitation of the RNA from the aqueous phase. The remaining phases were stored at 4degC for later DNA isolation. RNA precipitated from the aqueous phase samples was solubilized in DEPC-treated water. RNA concentrations were assessed using a NanoDrop 1000 (Thermo Scientific) and quality was assessed using a BioAnalyzer 2100 (Agilent). RNA Integrity numbers from the Bioanalyzer, an assessment of RNA quality, are reported in S2 Table.

All RNA samples were treated with DNase-I (Ambion, Turbo DNA-free DNase-I, Life Technologies). A total of 6-10ug of RNA was treated in a 50uL reaction. RNA (2ug) was transcribed to cDNA using the High Capacity RNA to cDNA kit (Applied Biosystems). Specific transcripts were quantified by qRT-PCR using pre-validated IDT PrimeTime qPCR primers and probes, or custom designed primer probe sets (Table 1). Specifics of the reaction mixes for Dnase-I treatment, reverse transcription, and qPCR are provided in S3, S4 and S5 Tables. Primers were designed to span exon junctions at the 3’ end of each targeted gene and to have high specificity (S6 Table). Primer probe pairs were analyzed by serial dilution against wild type cDNA to confirm high efficiency of the qRT-PCR reaction and these data are presented in S2 Fig and S7 Table.

**Table 1:** qRT-PCR Primers and Probes.

Name	Sequence and modifications	IDT Assay ID	Probe Purification Method
***Actb* Primer 1**	TGC TTG CTG ATC CAC ATC TG	Mm.PT.56a.33712250.g	HPLC
***Actb* Primer 2**	AGA TTA CTG CTC TGG CTC CTA		
***Actb* Probe**	/5TET/ACC GAT CCA /ZEN/CAC AGA GTA CTT GCG /3IABkFQ/		
**Arap1 Primer 1**	GGT GTC CCA GAG TCA GAA C	Mm.PT.56a.41291663.gs	HPLC
**Arap1 Primer 2**	AAT GAA ATG CGC CGG AGT		
**Arap1 Probe**	/56-FAM/CCC TTT CCC /ZEN/TTC TCC GCC ATG TC/3IABkFQ/		
**Dstn Primer 1**	TGA TCT ATG CAA GCT CGA AGG	Designed using IDT software	HPLC
**Dstn Primer 2**	CTT TTC AGC AAT ACA GGT CCG		
**Dstn Probe**	/56-FAM/CAT GAG TAT/ZEN/CAA GCA AAT GGG CCA GAA G/3IABkFQ/3'		
**LacZ Probe**	/6FAM/CGG CAT TTT CCG TGA CGT CTC GTT/TAMRA/	Designed using IDT software	HPLC
**LacZ Primer F**	ATC AGG ATA TG TGG CGG ATG A		
**LacZ Primer R**	TGA TTT GTG TAG TCG GTT TAT GCA		
**Lyplal1 Primer 1**	CAG CCT CCC ATA GAA AAT CCC	Mm.PT.56a.12727909	HPLC
**Lyplal1 Primer 2**	TGC CCA GAA CAC CTT GAA TC		
**Lyplal1 Probe**	/56-FAM/CAA TCC ACT /ZEN/GAG CAC TTG ACA CAT ACT ATC A/3IABkFQ/		
**Ninj1 Primer1**	GTT AAG AAA GTC CAG CTT GGC	Mm.PT.56a.43468197	HPLC
**Ninj1 Primer2**	CCT CAT CTC TAT CTC CCT CGT		
**Ninj1 Probe**	/56-FAM/AGC ACG CCC /ZEN/ACT CCT ATC TGC /3IABkFQ/		
**Rab32 Primer 1**	GCA AGC ATG TTT TCC ACT AGG	Mm.PT.56a.29895651	HPLC
**Rab32 Primer 2**	CAA CAG CCA GAG TCC TTC C		
**Rab32 Probe**	/56-FAM/ATA TAA ACA /ZEN/TCG ACG AGG CCA CCC G/3IABkFQ/		
**Rgcc Primer 1**	TCC TTG CTT CAC ATA CTT GCT	Mm.PT.56a.13340638	HPLC
**Rgcc Primer 2**	AAT TCT CCA ACC AAC TCC TCT C		
**Rgcc Probe**	/56-FAM/AGT GTC ACC /ZEN/TAA TTT GGC TTT CCG AGG /3IABkFQ/		

Triplicate PCR reactions for each gene, tissue and biological replicate were set-up in a laminar flow hood at room temperature with a master mix containing hot start *Taq*DNA polymerase. Amplification and qPCR measurements were performed using the Applied Biosystems 7900HT Fast Real-Time PCR System, v 2.4.1 software. Thermal cycling conditions were: 10 min at 95°C for initial denaturation; and 40 cycles of 15 sec at 95°C and 30 sec at 60°C. Each reaction contained 2 μL of template cDNA and a reaction master mix containing 2X Thermo Scientific Maxima Probe/ROX qPCR Master Mix (Thermo Scientific, USA), 500 nM of each primer and 250 nM of probe. All qPCR assays were run with appropriate controls including the Non-Template Control and minus RT control. The qRT-PCR experiments were designed and conformed to the MIQE guidelines as described by Bustin [20]. Data were analyzed using ΔΔCt method with *Actb* as the internal reference [21].

The qRT-PCR was performed with normalization to *Actb* gene expression and relative quantities of transcript were determined by the delta delta Ct method. Data are presented as the targeted gene, and LacZ reporter gene expression in each mutant tissue, relative to wild type control gene expression in the same tissue. The mRNA abundance of the native gene was comparable for the Silenced and Expressed groups with a delta Ct relative to *Actb* averaged across all genes and tissues of 5.5 for the Silenced group and 4.86 for the Expressed group.

### DNA Extraction, Bisulfite Treatment, PCR & Amplicon Sequencing

The general method for determining methylation status of CpGs in amplicons of bisulfite-treated DNA has been described and validated by Masser et al. [22]. DNA was extracted from the TRI-reagent interphase using Back Extraction Buffer (Life Technologies). Specifics of the Back Extraction mix are presented in S8 Table. After incubation and centrifugation, the aqueous phase containing DNA was removed and transferred to a clean 1.5mL tube. DNA was precipitated using isopropanol. The resulting DNA pellet was dissolved in TE Buffer (Sigma). Concentrations and quality of the DNA were assessed using a NanoDrop 1000. Genomic DNA (5ug) was treated with bisulfite using the MethylEasy Xceed kit (Human Genetic Signature, Australia) according to the manufacture’s protocol and eluted with 50uL of the elution buffer. Bisulfite conversion rates were confirmed to be >98% by analyzing the cytosine to thymine conversion for cytosines not in CpG motifs.

Amplification of bisulfite treated DNA was carried out using KAPA2G Robust HotStart DNA Polymerase (KAPA Biosystems). We used a semi-nested PCR to produce clean defined bands which were excised for library preparation and sequencing. Primers used for amplification of CpG Islands are listed in Table 2. For each CpG island, from 3 to 12 overlapping amplicons were sequenced. For LacZ methylation, a set of seven non-overlapping amplicons were used covering 67% of the coding sequence. The primers used for LacZ amplification are listed in Table 3. Each PCR reaction had unique conditions depending on the Tm’s of the primer sets. PCR products were separated by agarose gel electrophoresis, excised and DNA was purified using E&K Gel Purification kit (E&K Scientific).

**Table 2:** CpG Island Primers.

Primer	Sequence	Sequenced Amplicon length
Arap1-BS-F1	GTAAGTTAAGGATAAAGGAGAGATATAAAT	224
Arap1-BS-R2	CAACAACCTCACTAACCTATTTTAAAC	
Arap1-BS-R3	CCCCAAACAAAAACRCRCACCAAATAAC	344
Dstn-BS-F1	GGGTGGTTTTGTGGAAGTAGT	225
Dstn-BS-R1	CTACACAATTTTCCTCCTTCCTAAAAC	
Dstn-BS-F2	GGAATAAGYGTTGATTTATATAGTATTTTG	170
Dstn-BS-R2	CCRATAAACRAATACTCTACTTAAC	
Dstn-BS-F3	GGTTTTTGAGAGTYGTTTTGAGAGT	150
Dstn-BS-R3	CTCCRAAAAATCTAACCCTACCCTAAAAAC	
Dstn-BS-F4	GGTGGYGATTTTTATGTATGGGTAAAGGT	162
Dstn-BS-R4	CCCCACCCAAAAATCCCAATAATC	
Dstn-BS-F5	GGGGTTTTTAGTTGTTTGGAGATTATTG	230
Dstn-BS-R5	CRCCCGAAAATCACTCACCATATTC	
Lyplal1-BS-F3	GGAAGAGGATGAAGATGAGTGTTATATAGAAAG	173
Lyplal1-BS-R3	CCTCCRCCATAACTACTATACCCAAC	
Lyplal1-BS-F4	GGGTYGGAGATTAYGAGATTGTAATGTATTTGAGT	147
Lyplal1-BS-R4	CRCATACRCACACCCATACTAATACTACTTACCTAC	
Ninj1-BS-F1	GGGGATATTAAGGTYGTTGGAGAAGTGT	198
Ninj1-BS-R1	CTCATACTCCTCAATACCCRACTCCATAATAC	
Ninj1-BS-F2	GGGTGYGTTTAGGTYGTGTGTTG	177
Ninj1-BS-R2	CCRAACCAAACRCCCACAAATAC	
Ninj1-BS-F3	GTTTYGGTTGTTGTGTGATTTTGGT	162
Ninj1-BS-R3	CCCRACRCCTCTACCTACCTACTATACTAAC	
Ninj1-BS-F4	GTTTGATTTGTTTTTAGYGTAAGTGGTGGT	184
Ninj1-BS-R4	CCCRACRCCTCTACCTACCTACTATACTAAC	
Ninj1-BS-F5	GGTTGGTATTATTTTTYGGGTGGAGAAGGTT	156
Ninj1-BS-R5	CCCTTATCACTACTACAAACRACCGTATAATCAC	
Ninj1-BS-F6	GGTTGGAAGTTAGTATTAGGTAGATAG	198
Ninj1-BS-R6	CAAACATCCRACCATCRCTATAACCTTA	
Rab32-BS-F1	GTTTAGGTAGAGGAAAGGATAGAGAAGAAT	142
Rab32-BS-R1	CACCAACTCTTCTCCCAACACT	
Rab32-BS-F2	GAGYGTTTTGTTGTTTTAGTTGAGAATTTTG	181
Rab32-BS-R2	CCCTAAACCRTAACACCCATAAC	
Rab32-BS-F3	GAGTTYGTYGATGATTAGTATTTTAAAGAGGTGT	161
Rab32-BS-R3	CACCCAAACTATTCTAAACCAACAAC	
Rgcc-BS-F1	GAGTTTTAGGAAGTTTGAGTTTGAG	231
Rgcc-BS-R1	CCCCTCCAAATACCCTCTATAC	
Rgcc-BS-F2	GGTTTGTAGYGGTYGYGGAGTTTATG	178
Rgcc-BS-R2	CRCRTAAAATAAACTAAATAACTTCCAAACTTTC	
Rgcc-BS-F3	GGAGGAGAAGAAGTTTTYGTGGGTT	199
Rgcc-BS-R3	CTACCCACCCTTCTATCCTCCATC	
Rgcc-BS-F4	GGAGAYGGGATGTTAGGGTGATG	200
Rgcc-BS-R4	CCRCCCTCAACGCAAAACAATC	
Rgcc-BS-F5	GTAGAGGTGGAGAAGATATTTTTAAATAGGT	207
Rgcc-BS-R5	CCCCTACCRATAACTATAAAACGAAATAAC	
Rgcc-BS-F6	GAGTTTGAGYGTTAGTTATTYGTTTGAGT	234
Rgcc-BS-R6	CTCTAATACCCRCAAAAACACTTCCTTAAATC	

**Table 3:** LacZ primers used for Amplifying Reporter Gene Sequence.

Primer	Sequence	Size bp
**BS2-LacZ-F2**	GTTTTGTTTGGTTTTYGGTATTAGAAG	292
**BS2-LacZ-R2**	CCCRTTACACCACAAATAAAAC	
**BS2-LacZ-F4**	GTTTGTYGTTTGAATTTGATTTGAGYGCATTTTTA	284
**BS2-LacZ-R4**	CCCTACCATAAAAAAACTATTACCCRTAAATAATCAC	
**BS2-LacZ-F6**	GTTGATTGAAGTAGAAGTTTGYGATGT	315
**BS2-LacZ-R6**	ATCAAACRATTCATTAACACCATACC	
**BS2-LacZ-F8**	GAGTGTGATTATTTGGTYGTTGGGGAATGAATTAGGTTA	324
**BS2-LacZ-R8**	CCCAAACRAAACCRCCCTATAAACRAAAATACTAAC	
**BS2-LacZ-F10**	GGAAGTAAAATATTAGTAGTAGTTTTTTTAGT	290
**BS2-LaTZ-R10**	CTAATATACCCRACTTCTAACCATAC	
**BS2-LacZ-F12**	GAGTTGGGTAATAAGYGTTGGTAATTTAAT	259
**BS2-LacZ-R12**	AATCAACACCRCATCAACAAATATATCTA	
**BS2-LacZ-F14**	GGTAAATTGGTTYGGATTAGGGTYGTAAGAAAATT	294
**BS2-LacZ-R14**	CCRTCRATATTCAACCATATACCTTCTTC	

#### Library preparation for Next Generation Sequencing

Indexed libraries were prepared using TruSeq Sample Prep Kit Sets A/B (Illumina RS-122-2001/2) with the protocol started at the end-repair step with amplicons sonicated to 150-200nt length. Sequencing was completed either with the MiSeq Platform at the UC Davis Genome Center (150nt reads/paired end), or with the HiSeq2500 platform at the QB3-Berkeley Sequencing Core (single end 50nt). The number of reads per amplicon passing QC averaged 37,000.

#### Bioinformatics and Statistical Analysis of Methylation Calling

Sequencing reads were aligned to the MM9 genomic sequence using the Bowtie2 algorithm [23], and Bismark [24], was used for Bisulfite Indexed Genome creation and methylation calling. CpGs with reads <5 and assumed to be artifacts of PCR, and those CpGs falling into primer annealing sequence, were not included in the analysis. For each CpG, the proportion of methylated vs. non-methylated was calculated. Alignment files were converted to BAM format using SAMtools for visual inspection using the Integrated Genomic Viewer from the Broad Institute.

### Statistics

At each specific CpG site within a CGI, the significance of differences between the mutant and wild type allele were calculated with the non-parametric Fisher Exact test. For overall CGI differences in methylation, the mutant and controls were compared using the Cochran-Mantel-Haenszel statistic for categorical data. Comparing the Silenced and Expressed groups for percent CGI methylation, and for relative gene expression by ΔΔCt, we used a one-tailed t-test. Regression analysis was completed by the least mean squares method and an F statistic calculated to determine significance.

## Results & Discussion

**Fig 2:**
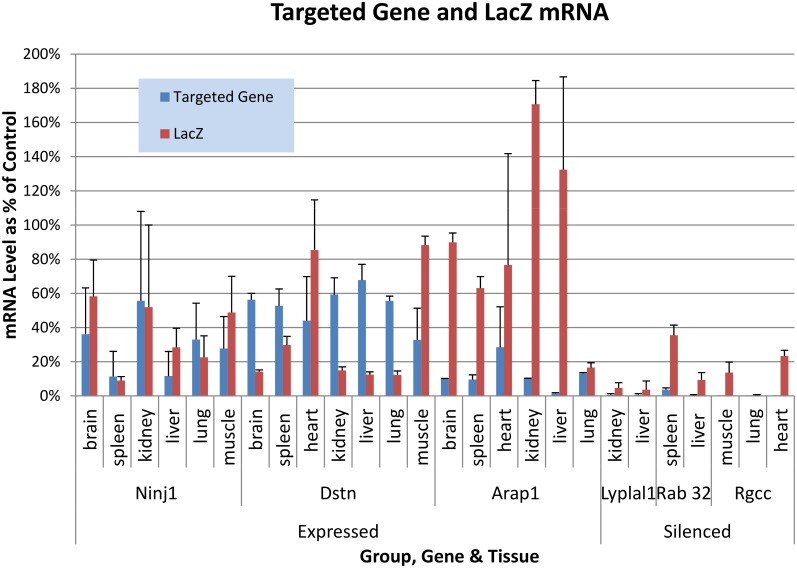
RQ mRNA of Targeted Genes and LacZ. Silenced and Expressed group targeted gene expression, and LacZ reporter gene expression, are presented as Relative Quantity (2^-ΔΔCt^) values relative to native gene expression in Wild Type Control animals for each mutant gene and tissue. Each data point is the average of three biological replicates, with the qPCR done in triplicate.

Overall, relative mRNA quantity for the targeted gene, and mRNA for LacZ, was less in the Silenced compared with the Expressed group (Fig 2). In the Silenced mutants (*Lyplal1*, *Rab32*, *Rgcc*), the targeted gene mRNA and LacZ mRNA was 0.6 ± 1.21% and 10.4 ± 11.6% of Wild Type Control native gene, whereas for the Expressed group of mutants (*Ninj1*, *Dstn*, *Arap1*) mRNA for the targeted gene and mRNA for LacZ were 32.9 ± 20.5% and 53.4 ± 43.9% of controls (all data are presented as mean ± standard deviation)

**Fig 3:**
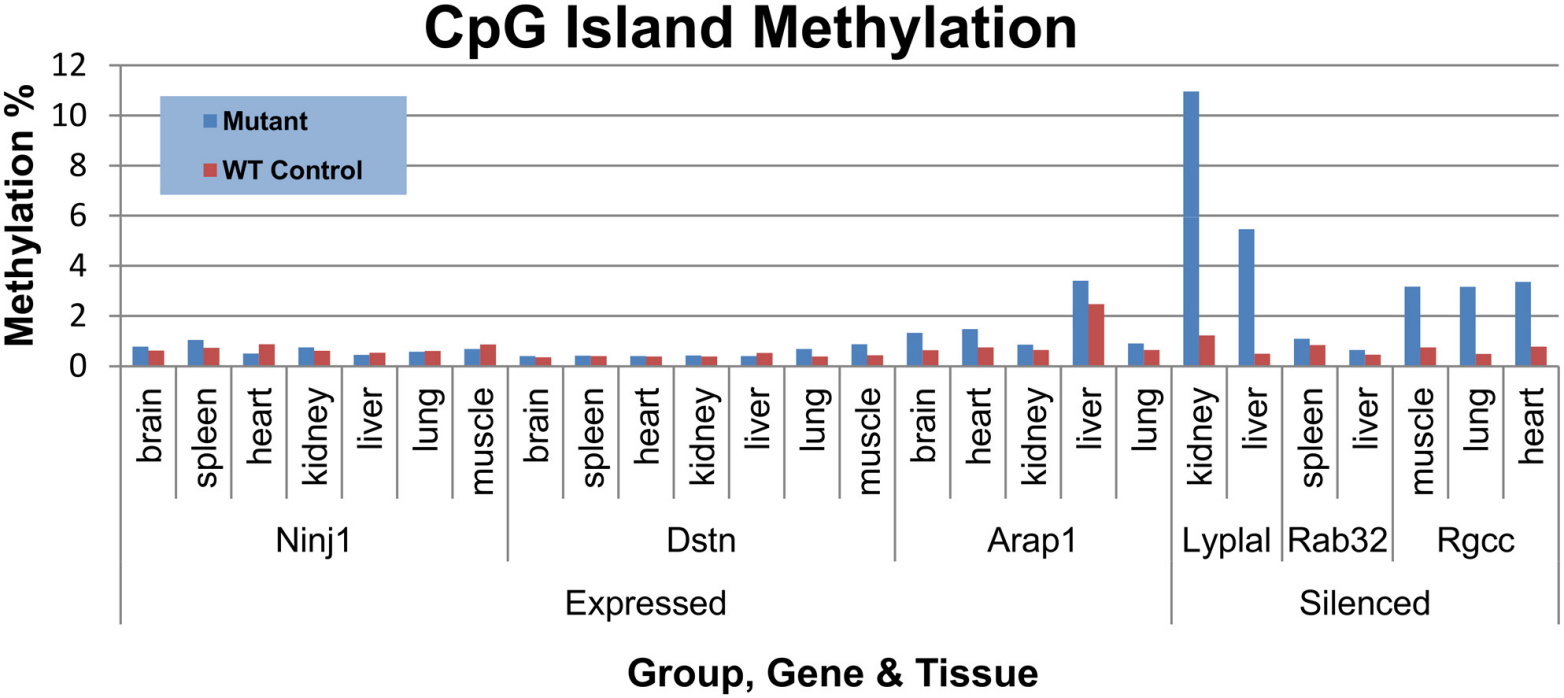
Methylation of CpG Islands in Targeted Genes. Percent methylation of CpG islands (CGI) in Mutant tissues and in Wild Type controls are presented for the Silenced and Expressed groups. Although the differences were small for some CGIs the Cochran-Mantel-Haenszel test determined that each mutant CpG island was significantly different from the corresponding Wild Type Control island percent methylation.

In wild type control animals, methylation of the CpG islands found in the promoters of genes characterized in both Silenced and Expressed groups was very low, 0.71 ± 0.25% and 0.66 ± 0.44% respectively. However, in the mutant Silenced group the average promoter methylation was 3.98 ± 3.21%, but promoter methylation remained very low in the Expressed group of mutants at 0.85 ± 0.67% (Fig 3).

The methylation data at each CpG site within each CGI was analyzed using a non-parametric test (Fisher Exact test). Since the average number of reads per amplicon was 37,000, this statistical method had the power to score as significant (p < 10^−6^) even very small differences in methylation. When the Cochran-Mantel-Haenszel test was used to compare Mutant versus Wild Type control there was a significant difference at p < 0.0001 for each island in every tissue. However, it was clear from inspection of the data (Fig 3) that there were differences in the overall percent methylation in the Silenced compared with the expressed group.

**Fig 4:**
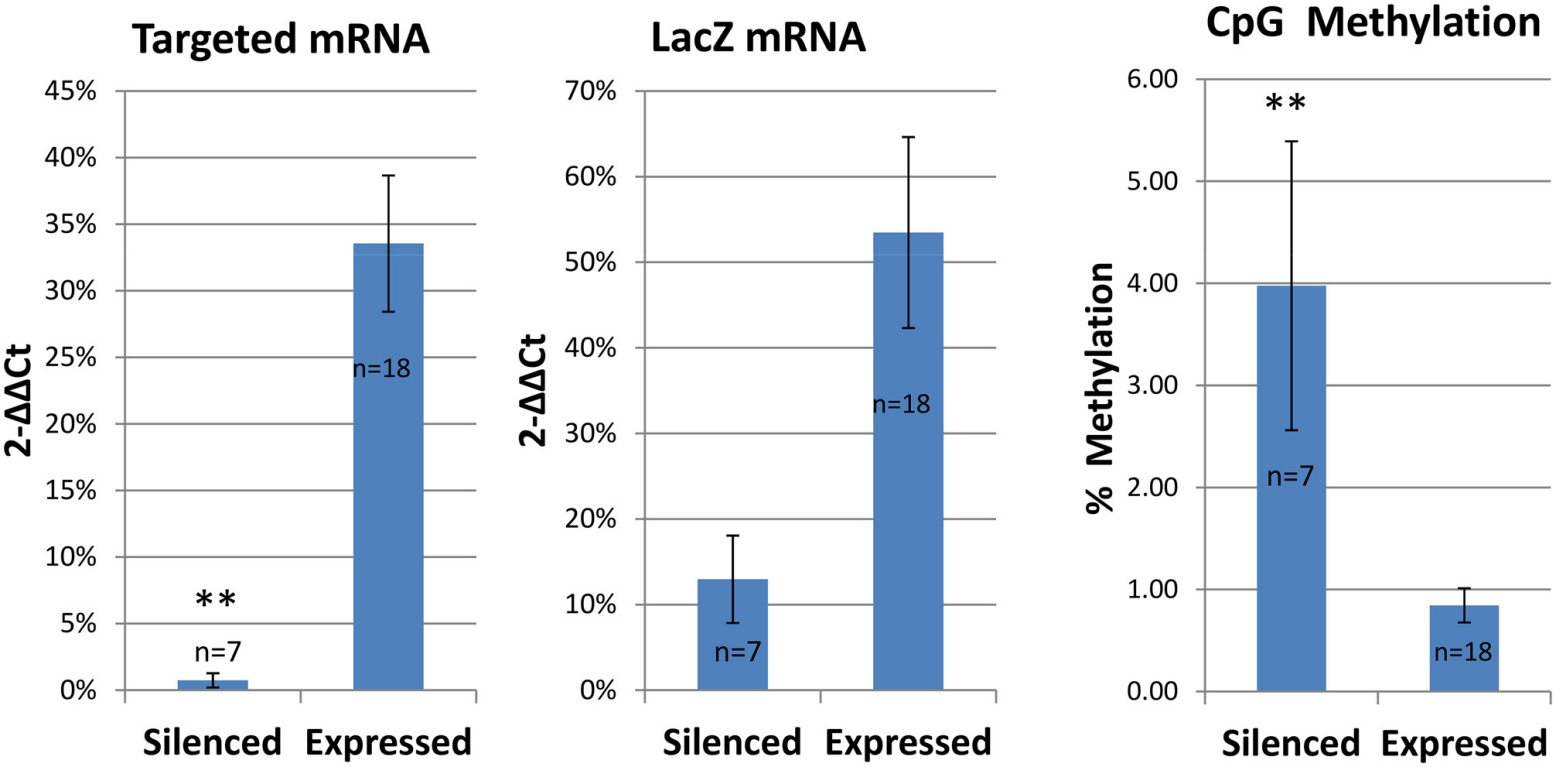
Targeted Gene Expression, Reporter Gene Expression and Promoter Methylation. Individual mutant and tissue values were averaged across the Silenced group (n = 7) and the Expressed group (n = 18) and compared by t-test. * = p<0.01; ** = p<0.0001. Date are presented as means +/- standard errors. The Silenced group had significantly lower expression of the targeted gene and the LacZ reporter and had higher percent methylation compared with the Expressed group.

The methylation and gene expression data for each group were averaged across mutants and tissues and compared by t-test and the data are presented in Fig 4. There were significant differences between the Silenced and Expressed group for the targeted gene mRNA abundance (p<0001), for LacZ mRNA (p<0.01), and significant differences for the percent methylation of the promoters (p<0.0001).

These data support the hypothesis that in a subset of targeted mutants carrying this allele there is a reduced expression of the reporter gene and the increased methylation of the promoters of the Silenced group supports the conclusion that this was due to promoter silencing. Not only was LacZ mRNA lower, mRNA for the targeted gene was significantly lower in Silenced compared with Expressed mutant tissue (Fig 4). The presence of mRNA for the gene-trap alleles is not surprising and likely represents splicing between the neo or LacZ reporter sequence and 3’ exons of the targeted gene, or leakiness of the gene-trap allele [25–28]. Of interest is that the quantity of targeted gene mRNA assessed by qRT-PCR was significantly lower in the Silenced compared with Expressed mutants.

As reviewed by Deaton & Bird [29] and by Illingworth and Bird [30], CpG islands (CGI) are regions of DNA rich in CpG sequences compared with the rest of the genome. CGIs are found in the promoter regions of ~70% of all genes. Generally, the cytosines are not methylated in these CGIs in either expressed or inactive genes, while there is a high percentage of CpGs methylated outside of CGIs. It is not known what protects promoter CGIs from methylation of the CpG cytosines but likely histone modifications and transcription factor binding are protective. However, when methylation of CpGs does occur within CGIs this is strongly correlated with the silencing of transcription. Methylation of CpGs may not be the initiating event of silencing but instead may mark chromatin modifications with cytosine methylation locking in and stabilizing the silenced state. CpG methylation is of significant importance during development [31], for X inactivation [32], imprinting [33], and is observed in silenced tumor suppressor genes in cancer tissue [34]. As noted in the introduction, CpG methylation has also been associated with the silencing of transgene promoters.

**Fig 5:**
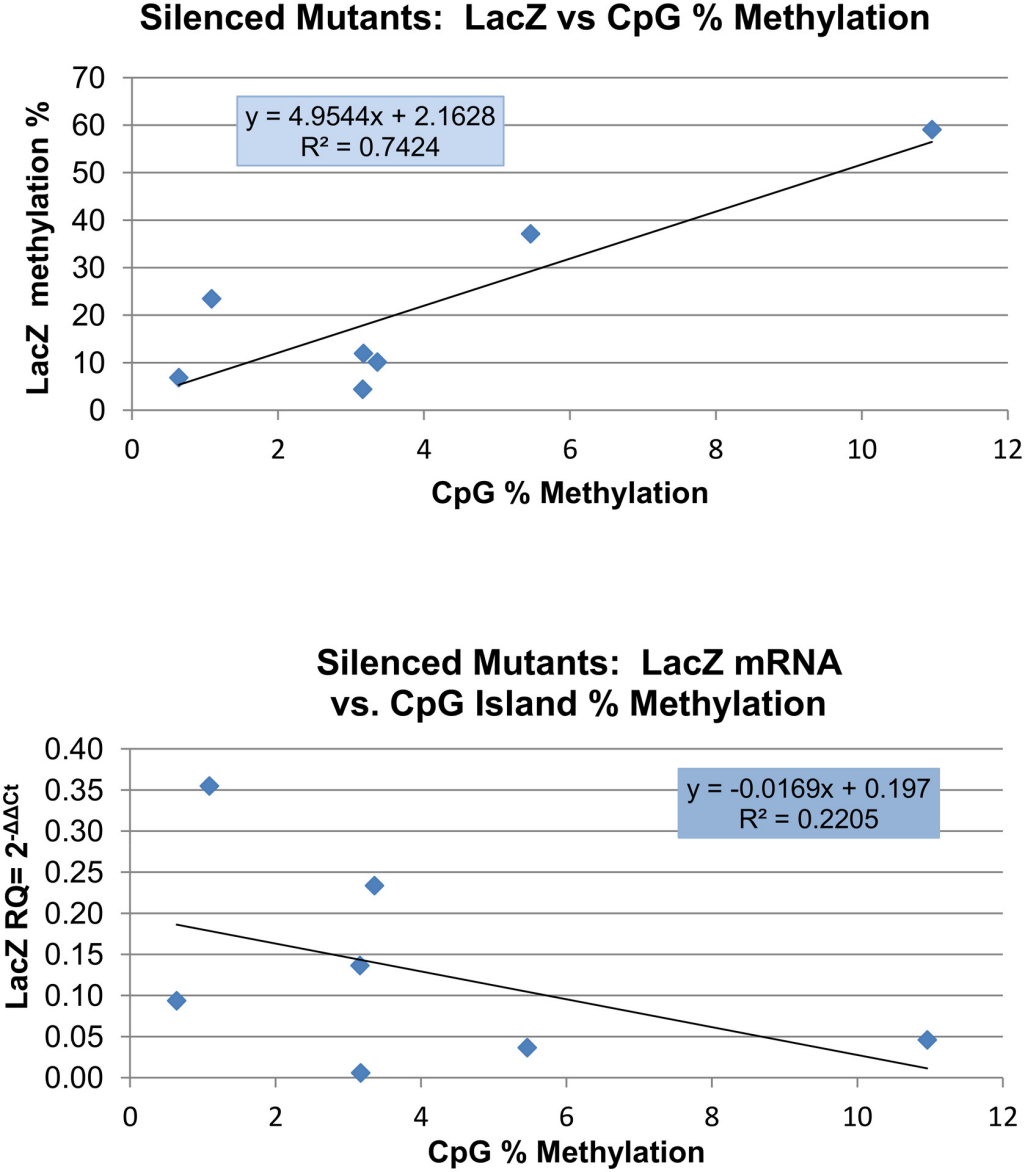
Correlation between Promoter and LacZ Coding Sequence Methylation, and between Promoter Methylation and LacZ Expression in Silenced Mutants. A significant positive correlation was observed between LacZ and CpG % methylation with a p < 0.013. Although the correlation between CpG% methylation and LacZ gene expression was negative as expected, this did not reach statistical significance.

We report here a small but statistically significant increase in CGI methylation in the promoters of targeted genes that did not have LacZ staining. This promoter methylation was correlated with LacZ methylation in the Silenced group (R^2^ = 0.74, p < 0.013; Fig 5). However, in the Silenced group there was only a weak and non-significant correlation of promoter methylation and LacZ mRNA (R^2^ = 0.22; Fig 5). This suggests that there are certainly other factors, in addition to promoter methylation, that are contributing to the silencing of gene expression in the Silenced group. As would be expected since the overall promoter methylation was uniformly very low in the Expressed group of mutants and tissues, there was no correlation between promoter and LacZ methylation, or between promoter methylation and LacZ mRNA.

Although the amount of overall methylation of the promoters in the Silenced group was elevated it was still quite low. However, the pattern of methylation may be the important factor in driving silencing and not the overall percent methylation at a CGI. There is precedent that methylation of specific CpG sites in promoter regions correlates with silencing. For example, Furst et al. [35] have reported that methylation status of a single CpG site in the promoter for the estrogen receptor alpha gene is correlated with transcriptional silencing. In a genome wide survey, Medvedeva [36] found that methylation of ~16% of CpGs in CGIs near the transcriptional start site were correlated with gene repression. However, these CpGs were generally not found at transcription factor binding sites. Of the three CGIs in the promoters of the Silenced group, only one had a uniform increase in CGI methylation across all the CpG sites in the mutant (*Lyplal1*). The other two CGIs had increased methylation in only a subset of CpGs. The patterns of CpG methylation for each of the silenced promoters in representative tissues are presented in S3 Fig.

**Fig 6:**
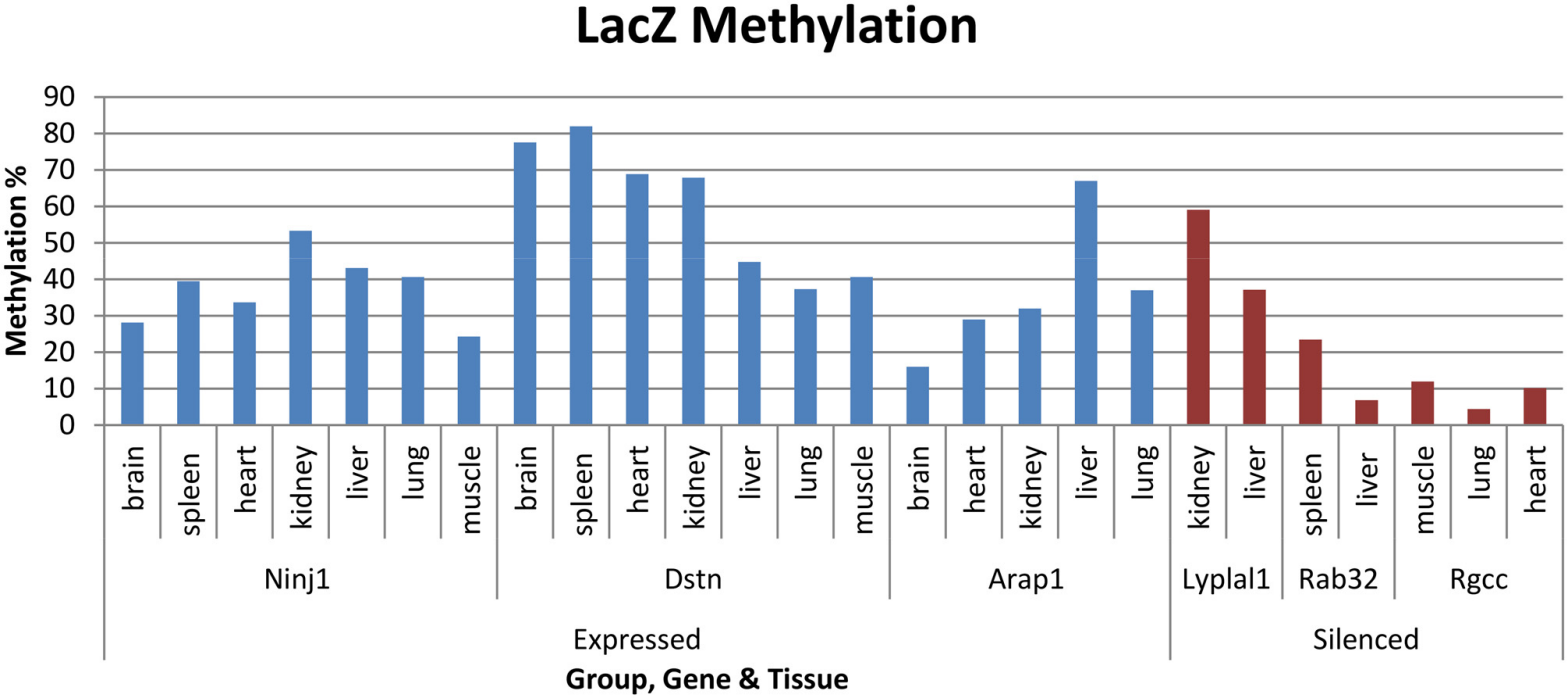
Methylation of CpGs in LacZ Coding Sequence. % methylation of CpGs in the LacZ coding sequence is presented for each mutant and tissue, with Silenced mutants in red and Expressed mutants in blue. There was a significant overall difference in average percent methylation of LacZ coding sequence between the two groups (t-test; p < 0.001).

LacZ was highly methylated for all of the mutant alleles and tissues (Fig 6). The percent methylation of LacZ CpGs in the Silenced group was 21.9 ± 18.5%, whereas the percent methylation in the Expressed group was 45.4 ± 18.3%. This was significantly different by t-test (p < 0.008). Gene bodies (exons and introns) are generally CpG poor relative to the rest of the genome, but the CpGs in gene bodies are highly methylated although the functional consequences of gene body methylation are not understood [37]. Gene body methylation does not block transcription or elongation [37]. In fact, there are reports that methylation of gene bodies is correlated with high gene expression on the active X chromosome [38]. Aran et al. [39] demonstrated that actively expressed gene bodies are hypermethylated compared with flanking sequences and compared with gene bodies that are not expressed. Whereas, Jjingo et al. [40] showed that gene bodies with the highest level of methylation are the genes with mid-level of expression, and genes with low and highest levels of expression have low methylation levels. Therefore, the relationship between exon/intron methylation and gene expression is not a simple one. One possibility is that the differential LacZ coding sequence methylation in the Expressed and Silenced group also contributed to the differential reporter gene expression in these two groups, with the higher methylation of LacZ coding sequence in the Expressed group leading to higher transcription. This may be one additional factor, along with promoter silencing, responsible for regulating reporter gene expression in this system but further work will be needed to determine this.

This study does not definitively prove that the presence of the LacZ reporter with a high CpG content is causal for the silencing of the targeted promoter in the Silenced mutants as would be suggested by the data from Chevalier-Mariette [12]. LacZ sequence has a higher GC content and more CpGs than most of the mammalian genome, containing 3061 nucleotides, a GC content of 56.3% with an observed CpG over expected CpG ratio of 1.19. The presence of unprotected CpGs in the LacZ coding sequence may recruit methylation factors which may extend their methylation activity to nearby promoter CGIs. However, these mutants also carried a Neo selection cassette which is high in GC% and CpG content. Neo coding sequence length is 794nts, the GC content is 59.9%, and the observed-to-expected ratio of CpGs is 1.03. A number of factors could be at play in determining if a promoter is silenced with this targeting vector, including the presence of the exogenous high CpG content DNA as well as unique characteristics of the targeted gene sequence and local chromatin environment. It is likely not simply the presence of the LacZ coding sequence in a permissive environment producing silencing.

We looked for unique features of the Silenced vs. the Expressed gene structure/organization, vector insertion, and histone modifications in mouse genome screens that might explain why the CSD targeting vector insertion results in silencing in some genes but not others. All of the genes targeted in this study contain multiple exons, and the size and location of the promoter CGI is similar among the mutants. The CGIs were of similar length, GC content, and ratio of observed to expected CpGs. The vector insertion site tended to be closer to the CGIs in the Silenced compared with the Expressed group, and two of the vector targeting arms overlapped with the CGIs in the Silenced group while none of the Expressed group vectors had CGI homology arm overlap. Overlapping vector arms could have resulted in disruption of the CGIs, but we found no differences in CGI sequence compared with the reference genome. We also found that two of the promoters in the Silenced group, and none of the promoters in the Expressed group, were associated with H3K27 histone trimethylation, a marker of Polycomb silencing [41]. These studies were done in wild type mouse tissues and the H3K27 methylation may indicate that these specific genes are predisposed to silencing. Other than these findings, we found no other differences in gene structure, vector characteristics, or histone marks that might explain differences in silencing between the Silenced and Expressed groups of mutants. However, the sample used is too small to make generalizations. Future studies with larger sets of genes, may reveal structural elements correlated with promoter silencing.

The focus of this report is the promoter silencing of the targeted gene in these mutants. However, the possibility exists that the targeting vector may influence the expression of genes in close proximity. This had been reported in other targeted mutant mouse models, either due to disruption of intragenic regulatory elements [42–43], or the presence of the heterologous promoter and neo sequence [44–46]. In a recent report in a targeted *Slc25a21* mutation using the same vector as described in the present report [47], they observed reduced expression of a nearby gene. Using an allelic series including the intact vector, and a vector with the neo cassette removed but retaining the LacZ reporter, they demonstrated that the presence of the Neo cassette was responsible for producing the phenotypes of dental and craniofacial abnormalities along with otitis media and hearing impairment. The presence of the Neo decreased the expression of the 3’ *Pax9* gene, and a previous publication of a mutation in mouse Pax9 recapitulated some of the same phenotypes. Examining the GEO mouse expression databases revealed expression in kidney, liver, spleen and other tissues for mouse Slc25a21. However, the Slc25a1 adult mutant has no staining for the LacZ reporter except in the testis (See: https://www.sanger.ac.uk/mouseportal/), which could be nonspecific or ectopic. Therefore, the down regulation of the neighboring *Pax9* gene in the *Slc25a21* mutant may also be associated with down regulation of the targeted promoter.

## Conclusions

In a subset of targeted gene trap mouse mutants, we have observed the apparent silencing of the targeted gene promoter reflected by reduced LacZ mRNA from the reporter gene. In this subset of mutants, the degree of LacZ methylation is significantly correlated with CGI methylation, but CGI methylation is only weakly negatively correlated with LacZ mRNA levels. The data support the hypothesis that presence of the exogenous DNA in the targeting vector, interacting with local chromatin environment, may lead to promoter silencing of the target and that this silencing is marked by CpG methylation. These findings emphasize the need to consider the local effects of targeting vectors on reporter gene expression, and possibly local effects on neighboring genes. Although we believe that local promoter silencing is a relatively rare event in mutants carrying this allele, additional work will be required to define the frequency of these events, and also to understand which features of the targeted gene environment, interacting with the vector, promote silencing.

## Supporting Information

S1 Fig Visual representation of CGI’s of Expressing and Silenced groups. CpG sites in each cartoon are represented by vertical lines. The number of nucleotides covered by the images is indicated in the upper right corner of each CpG plot. Flanking the actual CpG islands in these plots are 200nts not included in the island definition.
(TIF)

S2 Fig qRT-PCR Probe Efficiency for Actb, LacZ, Arap1, Dstn, Ninj, Rgcc, Lyplal1 and Rab32. Each plot point is the Ct value obtained using serious dilution of target cDNA. Efficiency values were measured using the Ct slope method constructing a plot of Ct vs. log cDNA dilution factor.
(TIF)

S3 Fig CpG Methylation Wild Type vs. Mutant. Methylation percent representation of individual CpG sites in the promoter regions of Silenced group.
(TIF)

S1 Table Mutants, Tissues, LacZ Staining, and Gene Expression. Columns under BioGPS and GEO indicate fluorescent intensity of the signal from probes for the specific gene transcripts. Numbers separated by slashes indicate values for different probe-sets, hyphenated numbers indicate range of values from different probe-sets.
(DOCX)

S2 Table Total RNA quality. Quality evaluation of pooled biological replicates of RNA samples with BioAnalyzer prior to reverse transcription to cDNA.
(DOCX)

S3 Table Conditions for DNAse Treatment (DOCX)

S4 Table Reverse Transcription. Reagents, their amounts and temperature cycling conditions used for reverse transcription.
(DOCX)

S5 Table qPCR Reaction Conditions. Reagents and their amounts for qRT-PCR reaction.
(DOCX)

S6 Table qRT-PCR probe design. Information for number of exons for all the genes investigated, NCBI accession numbers, in-*silico* specificity, primer/probe annealing location, length of an amplicon, and splice variants targeted.
(DOCX)

S7 Table Table of Primer/Probe Efficiencies. Efficiency values that were used to correct relative expression for genes of interest. Efficiencies were determined using the Ct slope method for which a plot of Ct vs. log cDNA dilution factor was constructed.
(DOCX)

S8 Table Back Extraction Buffer. Reagents and their amounts for DNA isolation from interphase and organic phase left after RNA isolation.
(DOCX)
