# Supplementary figures and images for: Reporter Gene Silencing in Targeted Mouse Mutants Is Associated with Promoter CpG Island Methylation
Source: PLoS One. 2015 Aug 14;10(8):e0134155. doi: 10.1371/journal.pone.0134155 (PMC4537176; doi:10.1371/journal.pone.0134155)

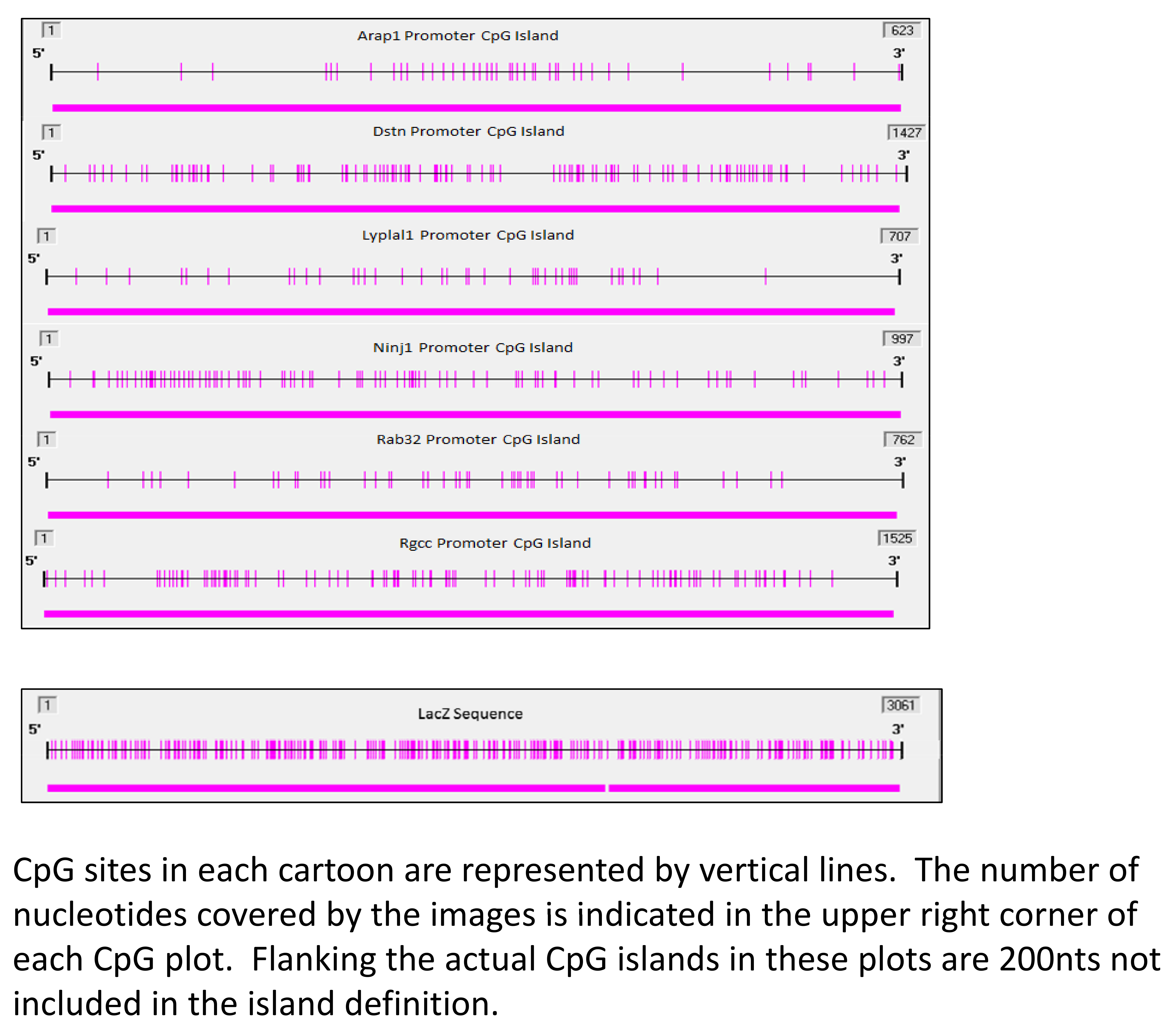

Supplement: S1 Fig — CpG sites in each cartoon are represented by vertical lines. The number of nucleotides covered by the images is indicated in the upper right corner of each CpG plot. Flanking the actual CpG islands in these plots are 200nts not included in the island definition. (TIF) [file pone.0134155.s001.tif]

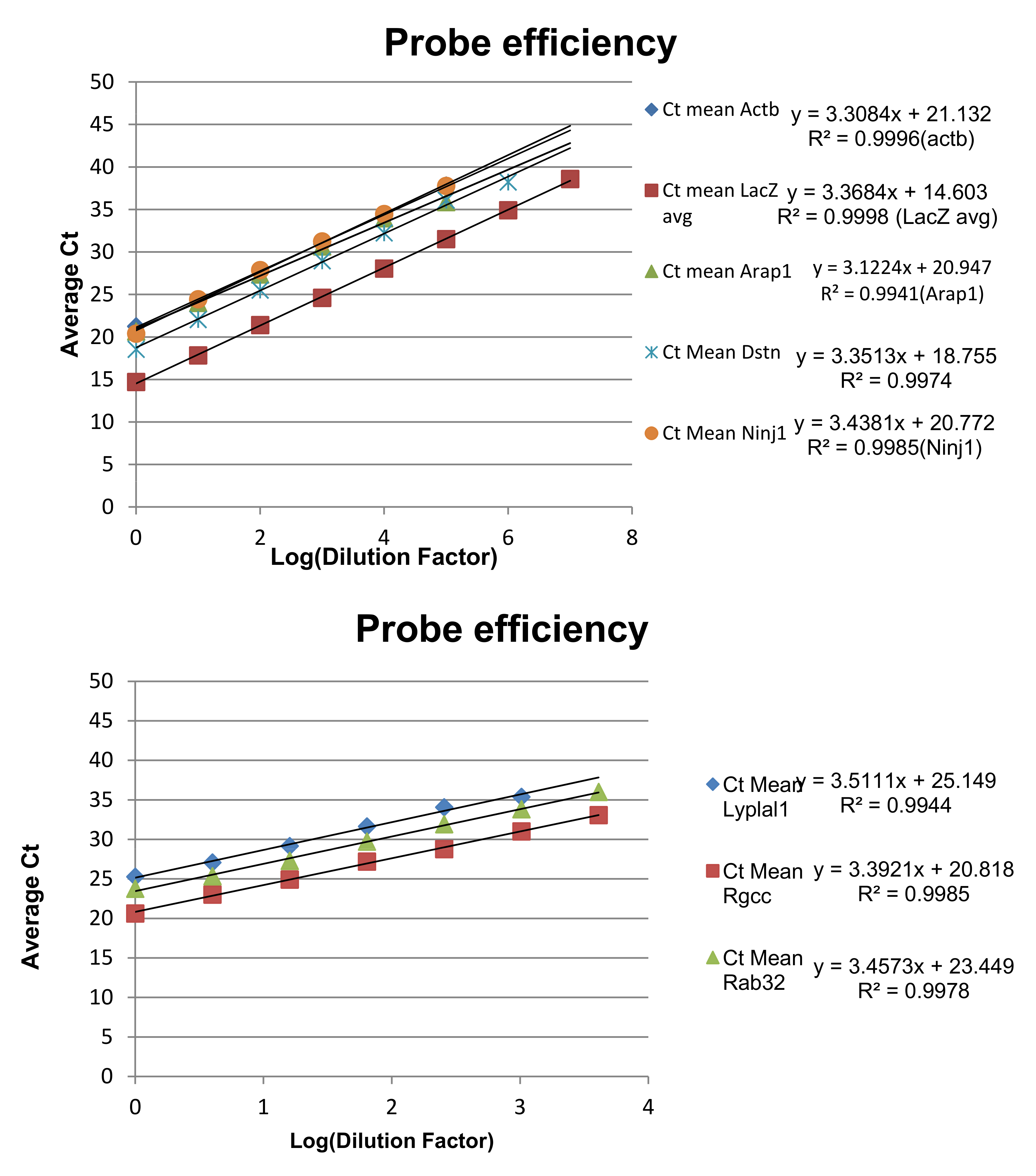

Supplement: S2 Fig — Each plot point is the Ct value obtained using serious dilution of target cDNA. Efficiency values were measured using the Ct slope method constructing a plot of Ct vs. log cDNA dilution factor. (TIF) [file pone.0134155.s002.tif]

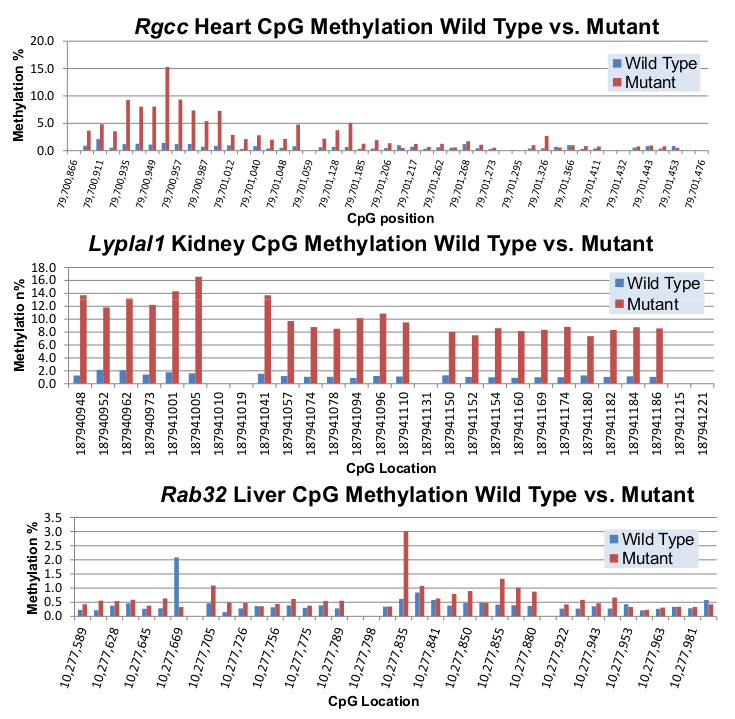

Supplement: S3 Fig — Methylation percent representation of individual CpG sites in the promoter regions of Silenced group. (TIF) [file pone.0134155.s003.tif]
