# Supplementary material for: Reporter Gene Silencing in Targeted Mouse Mutants Is Associated with Promoter CpG Island Methylation
Source: PLoS One. 2015 Aug 14;10(8):e0134155. doi: 10.1371/journal.pone.0134155 (PMC4537176; doi:10.1371/journal.pone.0134155)
Supplement: S1 Table — Columns under BioGPS and GEO indicate fluorescent intensity of the signal from probes for the specific gene transcripts. Numbers separated by slashes indicate values for different probe-sets, hyphenated numbers indicate range of values from different probe-sets. (DOCX) [file pone.0134155.s004.docx]

**Mutants, Tissues, LacZ Staining, and Gene Expression**

| **Gene ID** | **Tissue** | **KOMP LacZ Expression** | **BioGPS GeneAtlas MOE430 (FU)** | **BioGPS GeneAtlas GNF1M(FU)** | **GEO GDS 3052** |
| --- | --- | --- | --- | --- | --- |
| Arap1 | Brain | Staining | 42 | 66-96 | X |
|  | Lung | Staining | 141 | 171 | 338 |
|  | Spleen | Staining | 405 | 69 | 425 |
|  | Heart | Staining | 42 | 76 | 174 |
|  | Kidney | Staining | 34 | 68 | 187 |
|  | Liver | Staining | 105 | 67 | 201 |
| Dstn | Brain | Staining | 3,218-8,235 | 21,983-65,114 | 1,740 |
|  | Lung | Staining | 15,334 | 132,390 | 2,663 |
|  | Skeletal muscle | Staining | 2,371 | 9,489 | 652 |
|  | Heart | Staining | 7,083 | 34,931 | 2,180 |
|  | Spleen | Staining | 3,729 | 17,732 | 1,028 |
|  | Kidney | Staining | 8,729 | 63,721 | 2,252 |
|  | Liver | Staining | 6,153 | 38,998 | 1,742 |
| Lyplal1 | Kidney | No Staining | 1,385 | 950 | 518 |
|  | Liver | No Staining | 358 | 596 | 216 |
| Ninj1 | Brain | Staining | 88-441/47-212/12-41 | 811-1712 | 172 / X /299 / 763 |
|  | Lung | Staining | 444 /241 /35 | 1,801 | 145 / X /382 / 804 |
|  | Skeletal muscle | Staining | 198 /133 /45 | 1,967 | 339 /X /441 / 1028 |
|  | Heart | Staining | 1,061 /760 /124 | 1,679 | 260 /X /518 / 1122 |
|  | Spleen | Staining | 936/394/107 | 3,272 | 357/211/1029/720 |
|  | Kidney | Staining | 3,032 /2,085 /597 | 5,421 | 580 /353 /1215 / 1105 |
|  | Liver | Staining | 3,158 /3,117 /802 | 8,929 | 697 /351 /1659 / 1272 |
| Rab32 | Spleen | No Staining | 275 | 390 | 282 |
|  | Liver | No Staining | 558 | 2,686 | 194 |
| Rgcc | Lung | No Staining | 5,317/2,751 | 22,030 | 1343 / 2409 |
|  | Skeletal muscle | No Staining | 2,013/719 | 3,408 | 959 / 1809 |
|  | Heart | No Staining | 1,558/506 | 4,254 | 262 / 606 |

Columns under BioGPS and GEO indicate fluorescent intensity of the signal from probes for the specific gene transcripts. Numbers separated by slashes indicate values for different probe-sets, hyphenated numbers indicate range of values from different probe-sets.
