# Supplementary material for: Reporter Gene Silencing in Targeted Mouse Mutants Is Associated with Promoter CpG Island Methylation
Source: PLoS One. 2015 Aug 14;10(8):e0134155. doi: 10.1371/journal.pone.0134155 (PMC4537176; doi:10.1371/journal.pone.0134155)
Supplement: S2 Table — Quality evaluation of pooled biological replicates of RNA samples with BioAnalyzer prior to reverse transcription to cDNA. (DOCX) [file pone.0134155.s005.docx]

**RNA Quality**

| **KO** | **RNA pool** | **RIN** |  | **KO** | **RNA pool** | **RIN** |
| --- | --- | --- | --- | --- | --- | --- |
| Arap1 | Brain | 8.7 |  | Ninj1 | heart | 8.8 |
| Arap1 | spleen | 8.4 |  | Ninj1 | Sk ms | 8.8 |
| Arap1 | heart | 8.9 |  | Ninj1 | kidney | 9.2 |
| Arap1 | kidney | 8.3 |  | Ninj1 | liver | 9.3 |
| Arap1 | liver | 9.0 |  | Ninj1 | lung | 5.7 |
| Arap1 | lung | 7.9 |  | Rab32 | spleen | 7.9 |
| Dstn | Brain | 9.0 |  | Rab32 | liver | 9.2 |
| Dstn | spleen | 8.1 |  | Rgcc | Lung | 9.1 |
| Dstn | heart | 8.5 |  | Rgcc | Sk.ms | 8.8 |
| Dstn | Sk ms | 8.5 |  | Rgcc | Heart | 8.5 |
| Dstn | kidney | 9.0 |  | WT | Brain | 9.0 |
| Dstn | liver | 9.0 |  | WT | spleen | 8.4 |
| Dstn | lung | 8.6 |  | WT | heart | 8.3 |
| Lyplal1 | Liver | 8.9 |  | WT | Sk. Ms | 8.6 |
| Lyplal1 | Kidney | 9.0 |  | WT | kidney | 9.1 |
| Ninj1 | Brain | 9.1 |  | WT | liver | 9.2 |
| Ninj1 | spleen | 8.0 |  | WT | lung | 9.1 |
