# Supplementary material for: Reporter Gene Silencing in Targeted Mouse Mutants Is Associated with Promoter CpG Island Methylation
Source: PLoS One. 2015 Aug 14;10(8):e0134155. doi: 10.1371/journal.pone.0134155 (PMC4537176; doi:10.1371/journal.pone.0134155)
Supplement: S3 Table — (DOCX) [file pone.0134155.s006.docx]

**DNase-l treatment**

| **Components** | **Amount** |
| --- | --- |
| Total RNA | 6-10ug |
| 10X Turbo DNase Buffer | 5uL |
| RNase free water | to 50uL |
| Dnase I, RNase free | 1uL |

DNase-I treatment was completed at 37C for 60 min total. Reaction was terminated using stopping reagent provided with the Ambion Turbo DNase-I kit.
