# Supplementary material for: Reporter Gene Silencing in Targeted Mouse Mutants Is Associated with Promoter CpG Island Methylation
Source: PLoS One. 2015 Aug 14;10(8):e0134155. doi: 10.1371/journal.pone.0134155 (PMC4537176; doi:10.1371/journal.pone.0134155)
Supplement: S4 Table — Reagents, their amounts and temperature cycling conditions used for reverse transcription. (DOCX) [file pone.0134155.s007.docx]

**Reverse Transcription**

| **Components** | **Amount** |
| --- | --- |
| Total RNA | 500ng-2ug |
| 2X RT Buffer Mix (includes dNTPs, random octamers, and oligo dT-16) | 10uL |
| RNase free water | to 20uL |
| 20X Enzyme mix (MuLV and RNase inhibitor protein) | 1uL |

cDNA was synthesized at 37C for 60 min. Reaction was heat terminated at 95C for 5 min.
