# Supplementary material for: Reporter Gene Silencing in Targeted Mouse Mutants Is Associated with Promoter CpG Island Methylation
Source: PLoS One. 2015 Aug 14;10(8):e0134155. doi: 10.1371/journal.pone.0134155 (PMC4537176; doi:10.1371/journal.pone.0134155)
Supplement: S5 Table — Reagents and their amounts for qRT-PCR reaction. (DOCX) [file pone.0134155.s008.docx]

**qPCR Reaction Conditions**

| **Reaction components** | **Volume uL X1** |
| --- | --- |
| 2X MasterMix | 12.5 |
| H2O | 8 |
| Primers and probe | 2.5 |
| Template | 2 |
