# Supplementary material for: Reporter Gene Silencing in Targeted Mouse Mutants Is Associated with Promoter CpG Island Methylation
Source: PLoS One. 2015 Aug 14;10(8):e0134155. doi: 10.1371/journal.pone.0134155 (PMC4537176; doi:10.1371/journal.pone.0134155)
Supplement: S6 Table — Information for number of exons for all the genes investigated, NCBI accession numbers, in-silico specificity, primer/probe annealing location, length of an amplicon, and splice variants targeted. (DOCX) [file pone.0134155.s009.docx]

**qRT-PCR probe design**

| **Gene** | **# Exons** | **Sequence accession #** | ***In Silico* probe specificity E-score** | **Exon-exon Location** | **Splice variants targeted** | **Amplicon length bp** | **Targeted Exons** |
| --- | --- | --- | --- | --- | --- | --- | --- |
| ***Actb*** | 5 | NM_007393 | 1.00E-04 | 5-6 | All (1) | 133 | N/A |
| **Arap1** | 37 | NM_001040111 | 4.00E-04 | 36-37 | All (4) | 95 | exon 10 |
| **Dstn** | 4 | NM_019771 | 8.00E-07 | 3-4 | All (1) | 109 | 2nd exon |
| **LacZ** | N/A | N/A | no hits | N/A | All (1) | 71 | N/A |
| **Lyplal1** | 5 | NM_146106 | 2.00E-08 | 3-4 | All (1) | 124 | 2nd exon |
| **Ninj1** | 4 | NM_013610 | 0.005 | 3-4 | All (1) | 141 | 2nd exon |
| **Rab32** | 3 | NM_026405 | 3.00E-05 | 2-3 | All (1) | 131 | 2nd exon |
| **Rgcc** | 5 | NM_025427 | 3.00E-06 | 3-5 | All (1) | 137 | 2 and 3 deleted |
