# Supplementary material for: Reporter Gene Silencing in Targeted Mouse Mutants Is Associated with Promoter CpG Island Methylation
Source: PLoS One. 2015 Aug 14;10(8):e0134155. doi: 10.1371/journal.pone.0134155 (PMC4537176; doi:10.1371/journal.pone.0134155)
Supplement: S7 Table — Efficiency values that were used to correct relative expression for genes of interest. Efficiencies were determined using the Ct slope method for which a plot of Ct vs. log cDNA dilution factor was constructed. (DOCX) [file pone.0134155.s010.docx]

**Table of Primer/Probe Efficiencies**

| **Primer/Probe set** | **Efficiency** | **% Efficiency** |
| --- | --- | --- |
| LacZ | 1.981 | 98.097 |
| Rab32 | 1.955 | 95.469 |
| Lyplal1 | 1.927 | 92.669 |
| Rgcc | 1.972 | 97.153 |
| Dstn | 1.988 | 98.789 |
| Ninj1 | 1.954 | 95.370 |
| Arap1 | 1.996 | 99.635 |
| Actb | 2.006 | 100.568 |
