# Supplementary material for: Reporter Gene Silencing in Targeted Mouse Mutants Is Associated with Promoter CpG Island Methylation
Source: PLoS One. 2015 Aug 14;10(8):e0134155. doi: 10.1371/journal.pone.0134155 (PMC4537176; doi:10.1371/journal.pone.0134155)
Supplement: S8 Table — Reagents and their amounts for DNA isolation from interphase and organic phase left after RNA isolation. (DOCX) [file pone.0134155.s011.docx]

**Back Extraction Buffer**

| **Back Extraction Buffer for 100ml** |
| --- |
| 4 M guanidine thiocyanate (FW 118.2) (Amresco) |
| 50 mM sodium citrate (FW 294.1) |
| 1 M Tris (free base) Tris (FW 121.14) |

One ml of Trireagent was used in the extraction. After the phase containing RNA was removed, 500uL of back extraction buffer was added to the organic and interphase mixture. Samples mixed by inversion for 15 sec and incubated for 10 min at room temperature then centrifuged at 12,000 x g for 15 min at 4°C to separate the phases. Upper aqueous phase containing DNA transferred to a clean tube. DNA is precipitated from aqueous phase with 400 μl of isopropanol. Samples were mixed by inversion and incubated for 5 min at room temperature then centrifuged at 12,000 x g for 5 min at 4°C to pellet DNA. Wash the DNA pellet with 1 ml of 75% ethanol.
